# Cerium-doped calcium carbonate microparticles combined with low-intensity ultrasound for efficient sonodynamic therapy in body sculpting

**DOI:** 10.1186/s13036-025-00505-z

**Published:** 2025-04-28

**Authors:** Jhih-Ni Lin, Chih-Ying Chi, Yu-Ying Lin, Che-Yung Kuan, Chia-Tien Chang, Li-Ze Lin, I-Hsuan Yang, Feng-Huei Lin

**Affiliations:** 1 Institute of Biomedical Engineering, College of Medicine and College of Engineering, National Taiwan University, Taipei, 106319 Taiwan; 2 Institute of Biomedical Engineering and Nanomedicine, National Health Research Institutes, Miaoli County, Zhunan, 350401 Taiwan; 3 Cardiovascular and Mitochondrial Related Disease Research Center, Hualien Tzu Chi Hospital, Buddhist Tzu Chi Medical Foundation, Hualien, 970473 Taiwan; 4 Ph.D. Program in Tissue Engineering and Regenerative Medicine, National Chung Hsing University, Taichung, 402202 Taiwan; 5 Department of Materials Science and Engineering, National United University, Miaoli County, Miaoli City, 360301 Taiwan; 6 Department of Biomedical Science and Technology, National Sun Yat-sen University, No. 70, Lien-hai Rd, Kaohsiung, 804201 Taiwan

**Keywords:** Body sculpture, Calcium carbonate, Cerium, Low-intensity ultrasound, Reactive oxygen species

## Abstract

Excessive caloric intake and reduced energy expenditure contribute to obesity and localized fat accumulation, adversely affecting overall health. Despite advancements in obesity treatments, noninvasive approaches for targeted fat reduction remain limited. This study introduces a novel sonosensitizer microparticle, cerium-doped calcium carbonate (CaCO_3_:Ce), and evaluates its potential application in combination with low-intensity ultrasound (LIUS) for noninvasive body sculpting. CaCO_3_:Ce was synthesized via an environmentally friendly method, producing uniform 1.77 μm particles optimized for endocytosis. Energy-dispersive X-ray and X-ray photoelectron spectroscopy confirmed successful cerium doping. The particles demonstrated excellent biocompatibility and generated reactive oxygen species under LIUS exposure. Safety was validated through biochemical, hematological, and histological analyses in Sprague–Dawley rats. Animal experiments further revealed that CaCO_3_:Ce combined with ultrasound significantly reduced body weight growth rates, waistline measurements, and subcutaneous fat accumulation. These findings suggest that CaCO_3_:Ce, coupled with LIUS, offers a promising, noninvasive, and low-risk strategy for body sculpting, addressing limitations of current methodologies.

**Graphical abstract:**

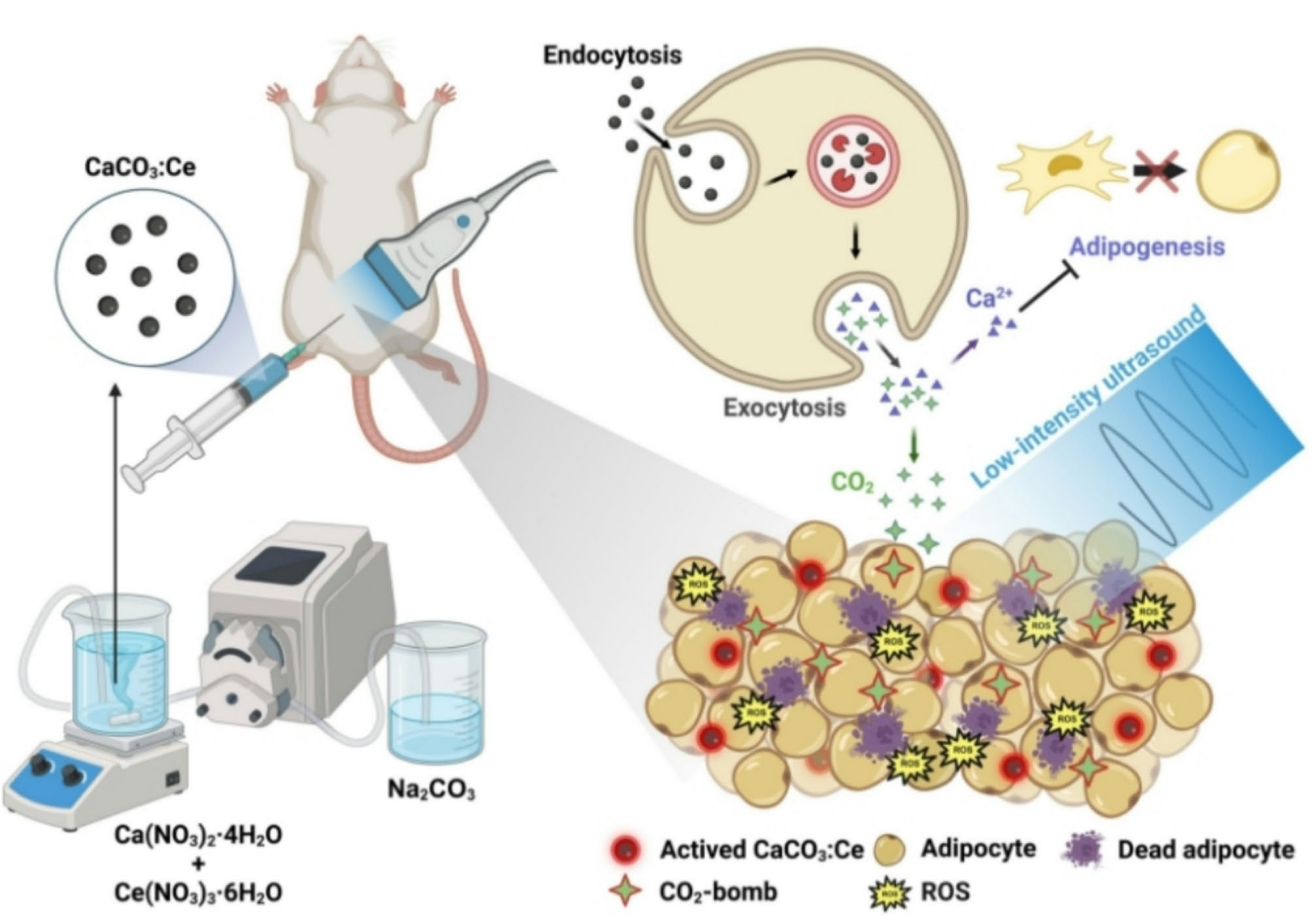

**Supplementary Information:**

The online version contains supplementary material available at 10.1186/s13036-025-00505-z.

## Introduction

An ideal body shape is not universal in humans [1]. On average, 50% of adolescent females are unhappy with their bodies, compared to 31% of adolescent males [2]. Increased caloric intake and reduced energy demands can lead to excessive body weight and localized fat. Excessive localized fat and body weight can impair an individual’s overall health because of multiple factors including physical, psychological, and genetic causes [3].

Over the past 25 years, obesity treatments have improved because of rapid advancements and the development of new approaches including bariatric surgery, intragastric balloons, lap bands, and numerous diets. However, only a few methods have been effective in successfully reducing localized adiposity [4]. Localized fat refers to adipose tissue concentrated in a specific area of the body, including the abdomen, flank, thigh, and upper arm [5]. Liposuction is a widely-used surgical technique for removing fat tissue from specific areas of the body to create a desired body contour, and is among the top five cosmetic surgical procedures performed in both men and women in the United States of America [6, 7]. However, this technique can result in various complications, including pulmonary embolism, lidocaine toxicity, infections, and even death [8, 9]. In addition to the safety concerns, several other factors have influenced the development of noninvasive or nonsurgical approaches for body contouring, which have gained popularity in recent years [6, 10, 11]. Noninvasive body sculpting is a rapidly growing field in cosmetic dermatology [12]. In 2018, the global body-sculpting market reached US$ 6.1 billion. Further, the body fat reduction market is expected to surpass $16.5 billion by 2025 [13].

Ultrasound (US) is not only a safe, portable, and non-invasive diagnostic tool for internal organ diagnosis but also a therapeutic modality [14]. Over the last decade, US has been developed as a commercial technique in plastic surgery for physical lipolysis. US used for body sculpting can be divided into two broad categories: low-intensity ultrasound (LIUS) and high-intensity focused ultrasound (HIFU) [15]. Among these, LIUS (0.5–17.5 W/cm^2^) can increase inertial cavitation and achieve bubble growth, which can be imploded to generate heat and stress that can destroy fat tissues to achieve lipolysis [16]. However, the results of breaking down fat tissue using LIUS have not been promising, and therefore, it can only be used as secondary treatment along with liposuction [17]. Alternatively, HIFU can burn down subcutaneous adipose tissue at high intensity (1000 W/cm^2^) with a special focusing plate to converge the ultrasonic waves to the intended ablation area without damaging the epidermis. In the targeted area, the focused energy induces a high temperature (> 65 ℃) to cause cell protein coagulation and disrupts the adipocyte membrane by a mechanical effect to contribute toward necrosis and apoptosis, thereby effectively dissipating adipose tissue. However, HIFU treatments can form hard subcutaneous nodules; cause discomfort, a burning sensation, and mild blisters; and char surrounding tissues, which can result in serious inflammatory responses [18].

Thus, although lipolysis performed using US is a good method for noninvasive and low-risk body sculpting, it has certain potential shortcomings that need to be addressed. In this study, we synthesized sonodynamic microparticles of rare element-doped calcium carbonate and used them in combination with LIUS for lipolysis to achieve mild and non-invasive body sculpting.

Calcium carbonate (CaCO_3_) has broad biomedical applications and is a potential candidate for the study because of its advantages such as safety, low cost, stability, and biodegradability. It exhibits sonoluminescence properties, wherein it absorbs energy from the explosion of ultrasonic cavitation to generate heat and react with oxygen or biomolecules for producing reactive oxygen species (ROS). The generated ROS can be converted into different free radicals to denature proteins for cell necrosis [19]. Further, CaCO_3_ can decompose into carbon dioxide (CO_2_) and calcium ions (Ca^2+^) in the acidic environment of the endosome–lysosome complex [20, 21]. The decomposed CO_2_ from CaCO_3_ can causes cell damage during explosive stress, further killing adipocytes [22]. In addition, Ca^2+^ released from the breakdown of CaCO_3_ in the endosome–lysosome complex increases the local calcium level around the adipose tissue, thereby inhibiting the differentiation of mesenchymal stem cells toward adipogenesis and pre-adipocyte maturation [23]. In our previous study, europium-doped CaCO_3_ combined with LIUS was used for local fat treatment without burning the skin or charring the tissues [24]. However, the body weight growth rate decreases by only ~ 20%, thereby limiting the efficacy of the sonodynamic particles in achieving body weight management. Nevertheless, the biocompatibility and side effects of sonodynamic particles when combined with LIUS can be improved by designing highly efficient sonoluminescent particles for clinical use.

Considering the superior biocompatibility and sonoluminescence properties of the rare element cerium (Ce), we envisioned doping Ce into the crystal lattice to partially substitute Ca^2+^ at the lattice site of CaCO_3_ to increase the sonoluminescent effect and clinical usability of CaCO_3_. To the best of our knowledge, this is the first study that employs this approach. In this study, we developed a method to synthesize Ce-doped CaCO_3_ (CaCO_3_:Ce) particles at relatively low temperatures without using organic solvents. We believe that the effective removal of local fat for body sculpting can be achieved by utilizing the free radicals produced from sonoluminescence, CO_2_-bombs, and localized increase in Ca^2+^ levels.

In this study, X-ray diffraction (XRD) was used to identify the crystal structure of the synthesized CaCO_3_:Ce. The XRD pattern was used to further examine the crystal structure of the individual synthesized grains using transmission electron microscopy (TEM). The morphology of the sonodynamic microparticles was observed using scanning electron microscopy (SEM). The semi-quantitative chemical compositions of the developed particles were examined and evaluated using energy-dispersive spectrometry (EDS). Further, X-ray photoelectron spectroscopy (XPS) was used to assess the electronic states of the elements present in CaCO_3_, and the particle size distribution was determined by dynamic light scattering (DLS) using a zetasizer. Water-soluble tetrazolium salt (WST-1) on L-929 cells were used to evaluate the cell viability of the developed material. Chloromethyl-2’,7’-dichlorofluorescein diacetate (CM-H_2_DCFDA) and live/dead stain were used to evaluate how the combination of CaCO_3_: Ce and LIUS works on 3T3-L1 cells; the results serve as the first screening in vitro. Finally, Sprague–Dawley (SD) rats were used as the target animals to evaluate the safety and efficacy in vivo, and their body weight, body temperature, waistline, weight of subcutaneous adipose tissue in the ultrasonic area, histological sectioning, blood element analysis, and serological analysis were measured and checked to prove the concept.

## Results

### Crystal structure identification

**Fig. 1:**
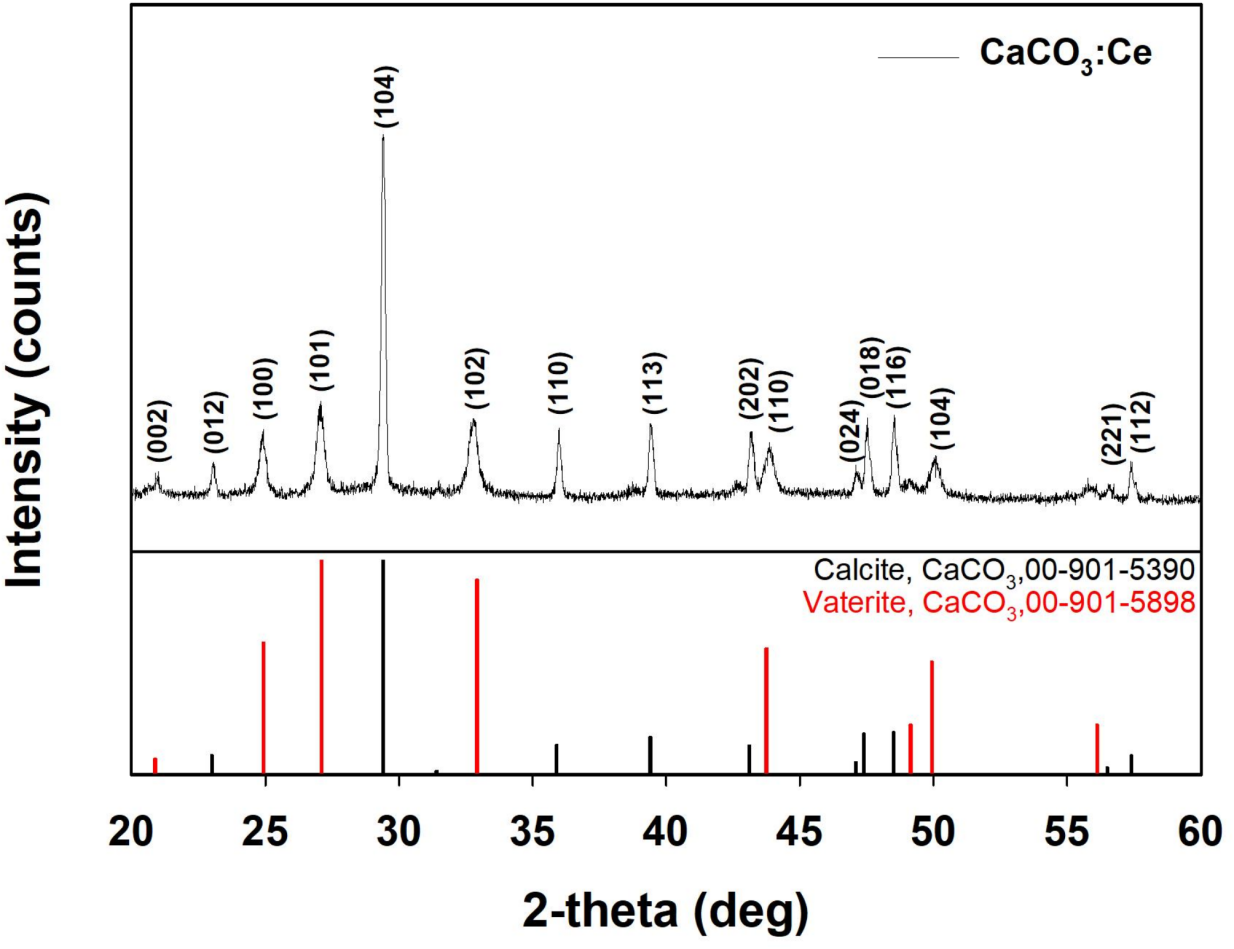
XRD pattern of CaCO_3_:Ce

The XRD patterns of the synthesized CaCO_3_:Ce are shown in Fig. 1. The characteristic peaks appeared at 2*θ* of 20.8°, 23.0°, 24.9°, 27.1°, 29.4°, 32.9°, 35.9°, 39.4°, 43.1°, 43.7°, 47.1°, 47.4°, 48.5°, 49.9°, 57.4°, and 58.0°, corresponding to the planes of (002), (012), (106), (101), (104), (102), (110), (113), (202), (110), (024), (018), (116), (104), (221) and (112), respectively. The peaks and relative intensities of the synthesized CaCO_3_:Ce were fully matched to those of calcite and vaterite CaCO_3_ as per the crystallography open databases No. 00-901-5390 and 00-901-5898, respectively.

**Fig. 2:**
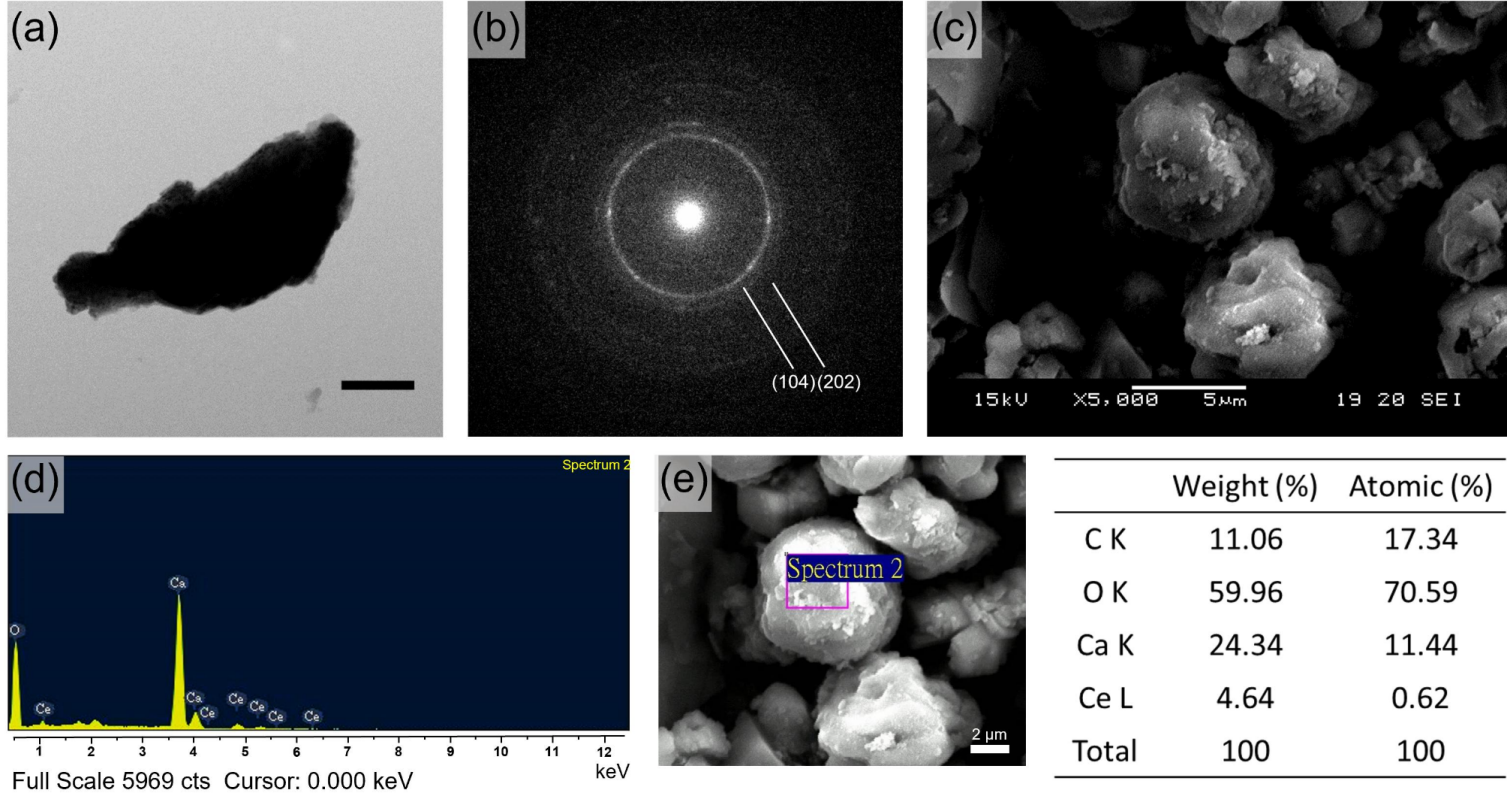
TEM image of (**a**) CaCO_3_:Ce, scale bar = 5 μm. (**b**) selected area electronic diffraction pattern of CaCO_3_:Ce. (**c**) SEM image of CaCO_3_:Ce. (**d**) Chemical composition and (**e**) the weight% and atomic percentage in average of each element of the synthesized CaCO_3_:Ce by EDS

The synthesized CaCO_3_:Ce was further examined using the TEM, and the selected electronic diffraction pattern (Fig. 2(b)) was a classic ring pattern, with d-spacings calculated from the ring pattern in agreement with the planes of (104) and (202) in the calcite crystal structure.

### Morphological examination and grain size evaluation

The surface morphology of the developed CaCO_3_:Ce was examined using SEM, as shown in Fig. 2(c). The image showed CaCO_3_:Ce present as cubic calcite and spherical vaterite, and it was aggregated into a particle with an approximate size of ~ 4 μm. The image was composed of many small rhombohedral grains stacked into particles. The nanosized grains formed a scalenohedron or prism, which can be seen from the edge of the TEM image in Fig. 2(a).

### Chemical composition analysis

The elemental composition of the synthesized CaCO_3_:Ce was determined by EDS to analyze the energy status of the electrons in different orbitals, as shown in Fig. 2(d). The major elements were carbon, oxygen, calcium, and cerium. The average weight percentages (weight%) and atomic percentages (atomic %) of each element are shown in Fig. 2(e). The results indicated that the substitution rate of cerium for calcium at the lattice sites was 4.64%.

### XPS analysis

**Fig. 3:**
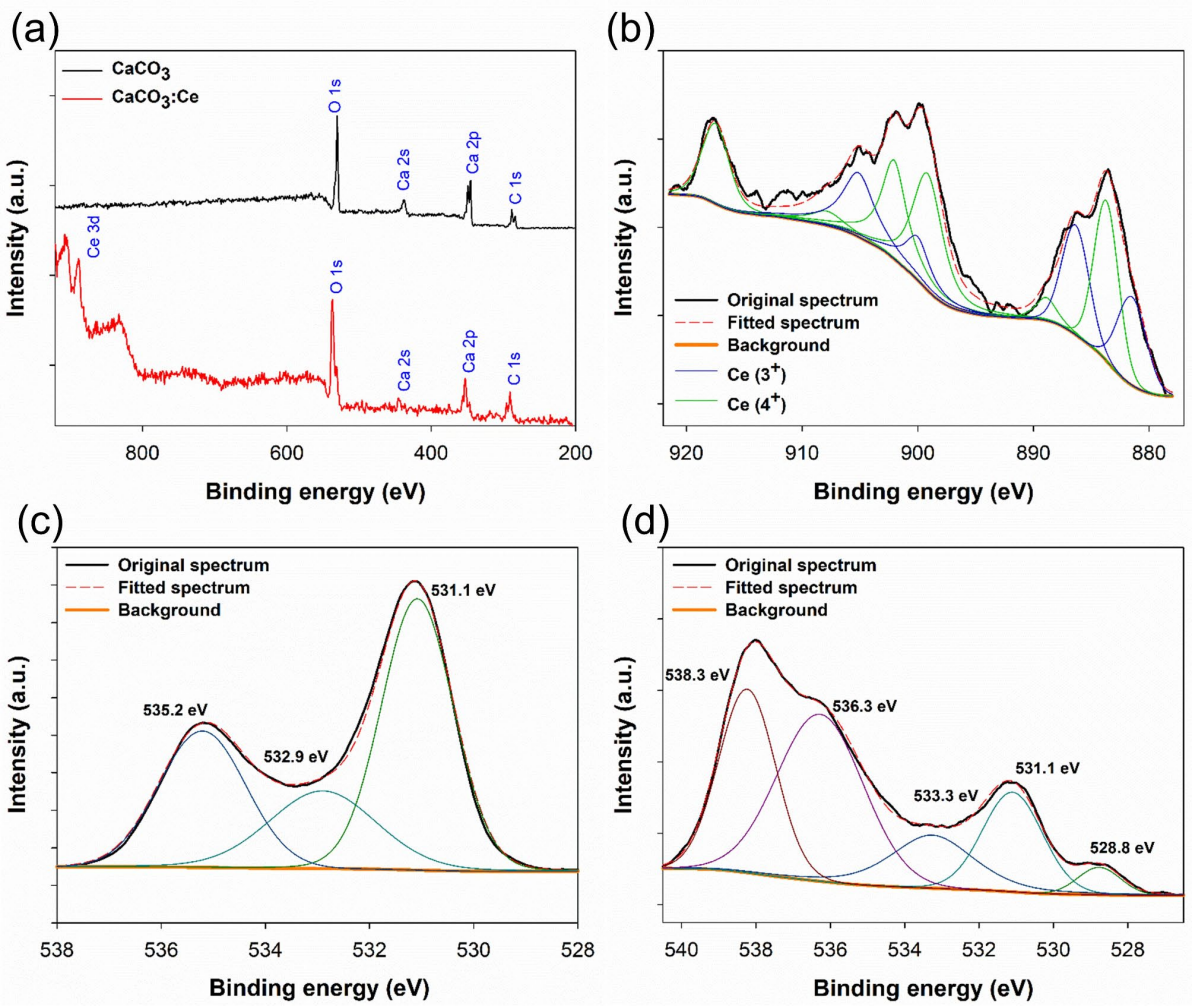
(**a**) XPS survey spectrum and high-resolution XPS spectra of (**b**) Ce 3d for CaCO_3_:Ce, (**c**) O 1s for CaCO_3_, and (**d**) O 1s for CaCO_3_:Ce

The surface survey XPS spectra of CaCO_3_ and CaCO_3_:Ce are shown in Fig. 3(a). The composites exhibited three major peaks corresponding to C 1s, Ca 2p, and O 1s in both groups, and additional peaks of Ce 3d (878–921 eV) were present only for the CaCO_3_:Ce group. Ce constituted 5.99% of all atomic proportions. Figure 3(b) shows the high-resolution XPS spectrum and curve fitting corresponding to the Ce 3d spectrum of the CaCO_3_:Ce group. The two and three spin–orbit doublet peaks (5/2 and 3/2) were attributed to the oxidation states of Ce^3+^ and Ce^4+^, respectively, confirming Ce doping into the CaCO_3_ structure. Based on these calculations, the relative total Ce concentration of Ce^3+^ was 39%, whereas that of Ce^4+^ was 61%. The O 1s spectra of CaCO_3_ and CaCO_3_:Ce are shown in Fig. 3(c-d), respectively. The O1s peak at a binding energy of 531.1 eV was assigned to Ca-O, whereas 532.9 and 533.3 eV were assigned to C-O on CaCO_3_ and CaCO_3_:Ce, respectively. However, the presence of the peak in both spectra at 535.2 and 536.3 eV were attributed to the multiplicity of the adsorbed water on CaCO_3_ and CaCO_3_:Ce, respectively. In Fig. 3(d), the binding energy of the O 1s peak at 528.8 eV corresponded to Ce-O of CaCO_3_:Ce. The peak at 528.8 eV corresponded to the absorbed oxygen in the CaCO_3_:Ce lattice. The high-resolution XPS profiles of Ca 2p and C 1s are shown in Figure S1.

### Evaluation of cytotoxicity in vitro

Figure S2 shows that the cell viability of the developed CaCO_3_:Ce followed the ISO 10993-5 standard. The cell viability of the control group, P-control, N-control, and experimental group of CaCO_3_:Ce were 100 ± 6.78, 8.38 ± 0.76, 96.14 ± 6.96, and 94.08 ± 3.23, respectively. The difference in viability between the control group and CaCO_3_:Ce was less than 25%. We conclude that the synthesized CaCO_3_:Ce did not induce cytotoxicity in L-929 cells and maintained normal cellular metabolism and mitochondrial function.

### ROS generation of CaCO_3_:Ce expose to ultrasonic irradiation

**Fig. 4:**
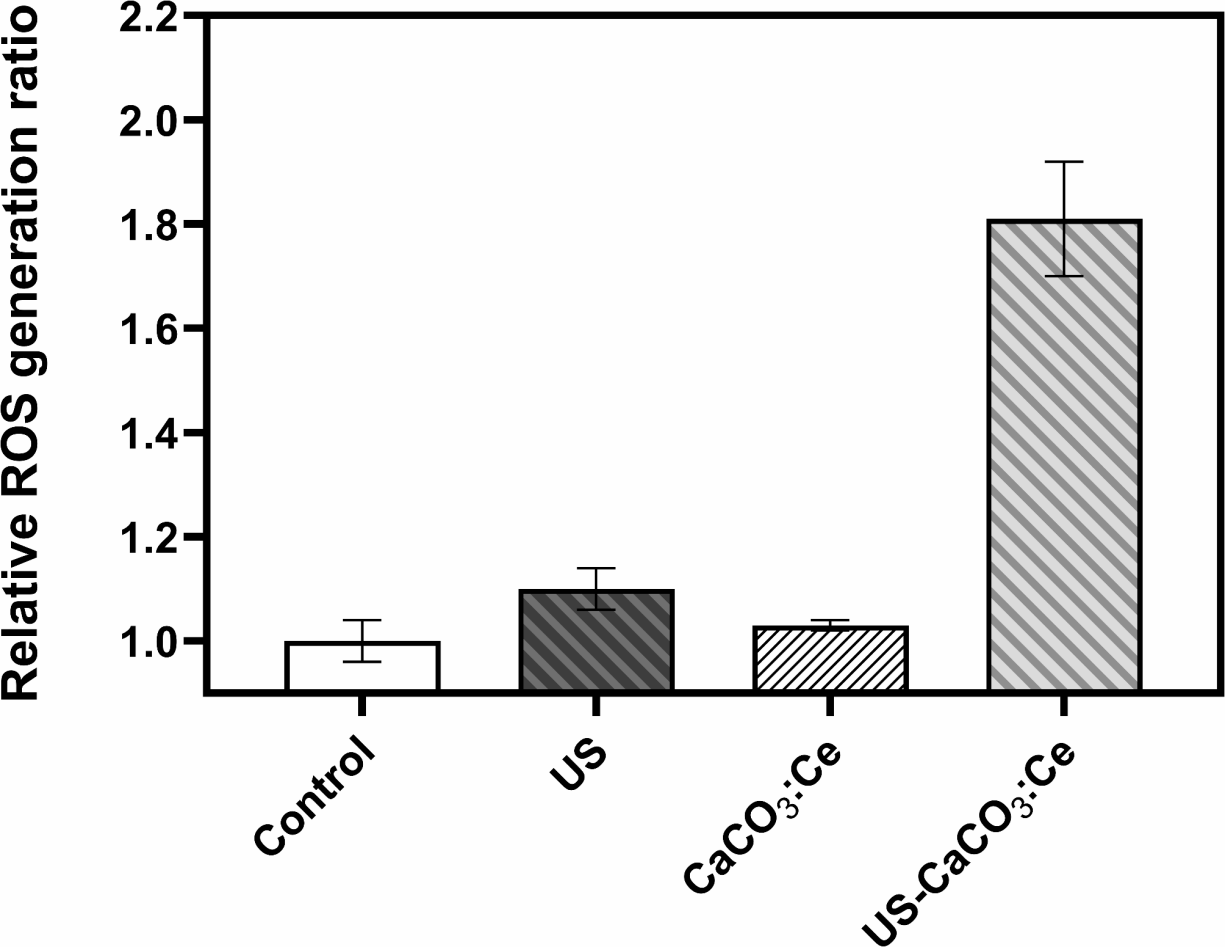
ROS production of 3T3-L1 cells treated with CaCO_3_:Ce with LIUS irradiation. *p < 0.05

Intracellular ROS production was measured using CM-H_2_DCFDA staining. The average fluorescence intensity of the control group was normalized to 1, and the values of the other groups were normalized based on the intensity of the control group as a relative value. The relative values were expressed in terms of relative ROS production. After 3T3-L1 cells uptake the developed CaCO_3_:Ce and are exposed to ultrasonic irradiation, the relative ROS production of the Control, US, CaCO_3_:Ce, and US-CaCO_3_:Ce groups were 1.00 ± 0.04, 1.10 ± 0.04, 1.03 ± 0.01, and 1.81 ± 0.11, respectively, as shown in Fig. 4. 3T3-L1 treated separately only by ultrasound irradiation (US) and the CaCO_3_:Ce particles (CaCO_3_:Ce) induced slight ROS generation, whereas cells treated with the combination of the synthesized CaCO_3_:Ce with LIUS (US-CaCO_3_:Ce) induced higher ROS generation.

### Efficacy of CaCO_3_:Ce exposed to ultrasound stimulation to induce adipocyte necrosis under ROS stress

**Fig. 5:**
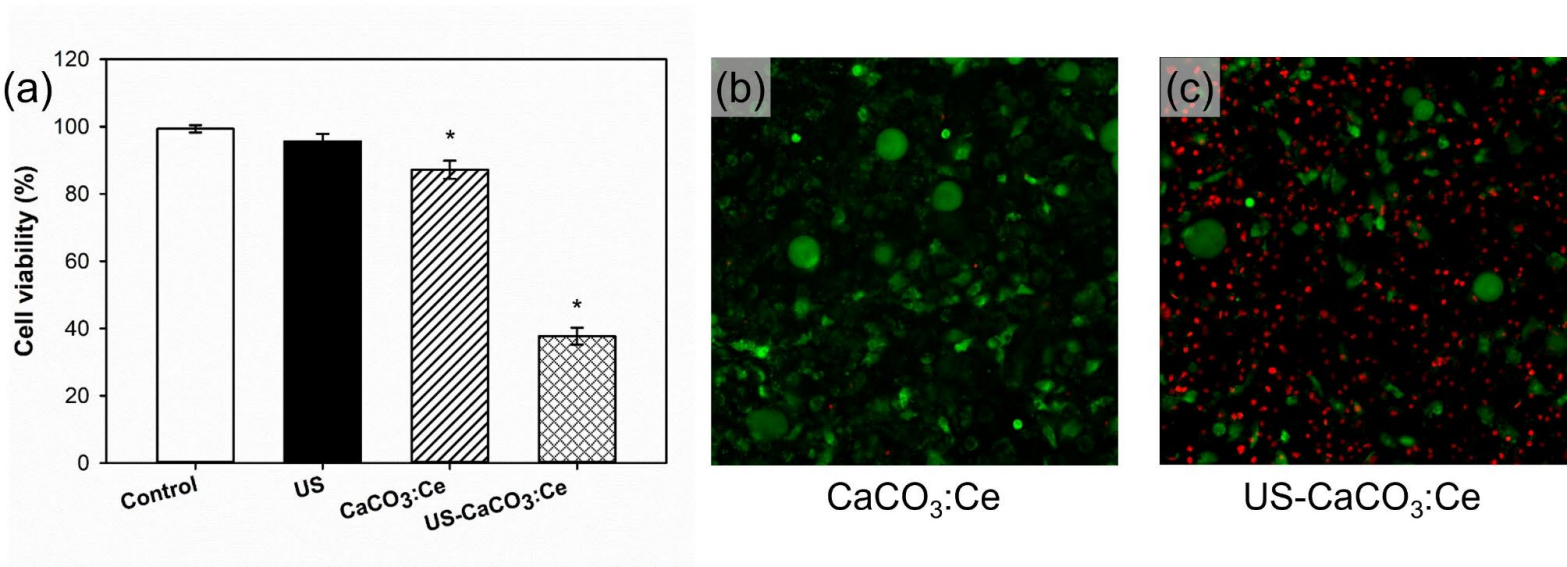
Cell viability of 3T3-L1 cells treated with LIUS, CaCO_3_:Ce, and a combination of CaCO_3_:Ce with LIUS stimulation, evaluated by (**a**) WST-1 assay, *p < 0.05, and live/dead staining of the (**b**) CaCO_3_:Ce and (**c**) US: CaCO_3_:Ce groups (scale bar: 100 μm)

The efficacy of CaCO_3_:Ce exposed to LIUS stimulation in inducing adipocyte necrosis under ROS stress was evaluated using the WST-1 assay and live/dead staining for determining the cell death rate. The cell viability was the same as in the previous description for normalizing the OD value to the control group as 1, and then, the values in the other groups were normalized to the control group to obtain a relative value. In Fig. 5(a), the adipocytes treated separately with ultrasonic irradiation (US) and CaCO_3_:Ce maintained mitochondrial function similar to the control group (control). Although CaCO_3_:Ce exhibited a slightly lower survival rate compared to the control group, the viability remained well above the 70% threshold defined by ISO 10993-5, indicating that CaCO_3_:Ce does not induce significant cytotoxicity. In contrast, mitochondrial function or cell viability was far less than that of the control group for cells treated with a combination of ultrasound irradiation and the developed CaCO_3_:Ce (US-CaCO_3_:Ce).

Figure 5(b-c) show that the cells treated with the combination of US and the developed particles had the highest death rate compared to that for the CaCO_3_:Ce group. Thus, the results of the WST-1 and live/dead staining indicate that the CaCO_3_:Ce combination with LIUS can effectively generate ROS to induce adipocyte death.

### Animal study

**Fig. 6:**
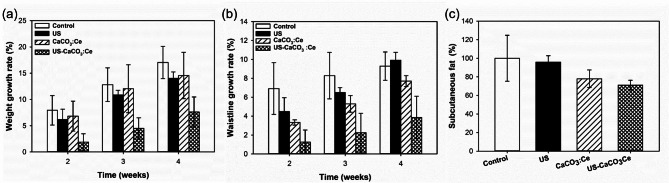
Weight growth rate (**a**), waistline growth rate (**b**), and subcutaneous fat percentages (**c**) of SD rats treated with US, CaCO_3_:Ce, and CaCO_3_:Ce with LIUS irradiation

Figure 6(a) shows the rate of body weight gain in rats injected with CaCO_3_:Ce in the abdominal area and then treated with LIUS. The body weight growth rate of rats without any treatment (control) was considerably higher than that in rats treated with a combination of CaCO_3_:Ce injection and LIUS irradiation (US-CaCO_3_:Ce). The growth rates in the control group were 7.93, 12.82, and 17.04 and that for the US- CaCO_3_:Ce group were 1.89, 4.49, and 7.62 at weeks 2, 3, and 4, respectively.

Figure 6(b) shows the waistline measurements of the experimental rats. The waistline of the rats treated with the combination of the developed particles and LIUS was considerably lower than that of the control group. The growth rate of the waistline for the combination treatment group was ~ 1.26, 2.25, and 3.86 in weeks 2, 3, and 4, respectively. This trend is identical to that observed for the increase in body weight.

The rate of subcutaneous fat is shown in Fig. 6(c). The growth rate of subcutaneous fat in rats treated with the combination of CaCO_3_:Ce injection and US stimulation at week 4 was 71.04% compared to that in the control group (100%). The results showed that the combination treatment effectively inhibited the growth rates of body weight, waistline, and subcutaneous fat.

## Discussion

Media, TV advertisements, and magazines display images to encourage people toward different ideal body shapes compared to their own phenotypes. An ideal body shape is not universal among human beings, and it depends on personal preferences. In addition, extreme methods used in the pursuit of an ideal body figure have been associated with eating disorders, depressive symptoms, anxiety, and stress, which may lead to irrational weight loss [1]. Therefore, a safe and effective method for body sculpting is required.

Currently, FDA-approved devices for body sculpting include cryolipolysis, lasers, high-intensity focused electromagnetic fields (HIFEM), radiofrequency (RF), and HIFU [12]. HIFU is a recent addition to non-invasive body-contouring tools, and it has gained popularity for visible skin tightening and rejuvenation. It utilizes acoustic energy to generate heat and induce apoptosis [28]. However, HIFU causes hard subcutaneous nodules, discomfort, burning sensations, mild blisters, and charred surrounding tissues, which can lead to severe inflammatory responses [18]. Conversely, LIUS mitigates these side effects using low-intensity US; however, its limited effectiveness allows it to function only as a supportive method along with liposuction.

Sonodynamic therapy (SDT) is a noninvasive therapeutic modality that combines LIUS and sonosensitizers. The key effect of US is the induction of nucleation, growth, and implosion of bubbles in the tissue, a phenomenon referred to as the cavitation effect [29]. Cavitation can be categorized into stable and inertial cavitation based on the way the bubbles collapse, depending on the intensity of the US irradiation. Under low sound pressure (< 0.1 MPa), bubbles exhibit stable cavitation, characterized by the periodic shrinkage and expansion of gas bubbles. Inertial cavitation is characterized by the bubbles collapsing instantly under sufficiently high sound pressure. Inertial cavitation can generate acoustic emissions, microstreaming, jetting, and shock waves, leading to mechanical damage to the target tissue. In addition, cavitation can produce ROS through the thermal dissociation of water; however, the yield of ROS generated by inertial cavitation under US irradiation is insufficient to achieve a strong therapeutic effect. Therefore, various sonosensitizers have been used for increasing the ROS concentration in tissues [30, 31]. SDT represents a safe, convenient, and feasible clinical treatment option with broad applications across various medical fields, including the treatment of cancer, neurodegenerative diseases, and bacterial infections [32, 33]. Therefore, our research focused on developing a combination therapy using LIUS with rare-earth element-doped sonodynamic particles for improving effectiveness and preventing the side effects caused by high-energy treatment.

Owing to their excellent biocompatibility and biological effects, rare-earth elements are now widely used in biomedical applications, including biometrics, bioimaging, drug delivery, and in the diagnosis and treatment of diseases [34]. In a previous study, experiments were conducted using Eu-doped calcium carbonate (CaCO_3_:Eu) and LIUS for localized body sculpting. The experimental results confirmed that the injection of acoustic sensitive materials in animals and the application of US in rats were safe, with no adverse effects on the physiological condition or organs of the rats. US irradiation in combination with CaCO_3_:Eu in SD rats led to a significant reduction in body weight, waistline, and subcutaneous adipose tissue. In summary, rare-earth element-doped sonodynamic treatment demonstrates significant potential for applications in body sculpting. Despite the positive effects of this material, Eu has limited applications in the biomedical field, and there is scope for improvement in the efficacy of body sculpting. Therefore, in this study, Ce was used as a replacement for Eu in the development of next generation sonodynamic CaCO_3_.

Ce is the most abundant element in the rare-earth family [35] and constitutes ~ 0.0046% of the Earth’s crust [36]. Ce^3+^ and Ce^4+^ are the two oxidation states of Ce [37], and they exert dual effects toward ROS generation and ROS scavenging, depending on their ratio in nanoparticles [38]. A higher Ce^4+^/Ce^3+^ ratio results in catalase-like activity. One study suggested that the Ce^3+^/Ce^4+^ ratio of cerium oxide-engineered nanoparticles can range between 1/4 and 1/2, depending on environmental factors and the aging of the nanoparticles. One study confirmed that a lower Ce^3+^/Ce^4+^ ratio can exhibit catalase-like activity [39]. In this study, the Ce^3+^/Ce^4+^ ratio of the developed CaCO_3_:Ce was 1/1.56, indicating that this material possesses good oxidation capability to produce ROS. We used a 2-hydroxyterephthalic acid (HTA) reagent for testing to verify whether ultrasound parameters with the developed particles can induce inertial cavitation in a solution containing the material. The reaction between terephthalic acid, which was used as the detection reagent, and hydroxyl radicals produced by inertial cavitation in water, resulted in the formation of fluorescent HTA. The test results were used to compare the background fluorescence of CaCO_3_:Ce and CaCO_3_:Eu without US set at 100%, with the respective groups subjected to US. The study synthesizing CaCO_3_:Ce revealed that the group with US exhibited higher HTA production, which was 2.65 times higher than that of the group without US, whereas for CaCO_3_:Eu, the ultrasound-exposed group showed higher HTA production (2.05 times higher) than that of the group without ultrasound (Figure S3). The results confirmed that the particles synthesized in this study, when excited by US, can effectively produce free radicals.

The synthesis of CaCO_3_:Ce did not require high temperatures or organic solvents, making it an environmentally friendly preparation method. The crystal structure was identified using XRD and matched the standard patterns of calcite and vaterite in CaCO_3_ (Fig. 1). A Zetasizer was used to analyze the size distribution of the synthesized particles (Figure S4). Aggregation bias was minimized by ultrasonic homogenization. The obtained polydispersity index (PDI) was 0.22, indicating a relatively uniform particle size distribution and suggesting that ultrasonic dispersion primarily reduces aggregation rather than causing structural damage to the particles. The average particle size of CaCO_3_:Ce was 1.77 μm, which falls within the optimal size range for cellular endocytosis (0.5–10 μm) [40, 41]. That said, we acknowledge that DLS alone cannot directly confirm potential surface modifications. Future studies incorporating proper analyses (e.g. SEM/TEM) before and after ultrasonic treatment would be valuable for further verifying the stability of the microparticles. EDS and XPS analyses confirmed that Ce was successfully doped into the CaCO_3_ lattice at a rate of ~ 5%. In Fig. 5(d), the peak at 528.8 eV indicates oxygen adsorption on CaCO_3_:Ce, which can be attributed to crystal defects introduced during the Ce doping process [42].

In the in vitro experiments, analysis using WST-1 confirmed that CaCO_3_:Ce exhibited excellent biocompatibility (Figure S2). Furthermore, the combination of CaCO_3_:Ce and LIUS interacted with differentiated adipocytes (3T3-L1) and was expected to effectively inhibit the cellular activity of 3T3-L1 (> 50%) (Fig. 5). In the animal study, as the rats continued to grow, all groups showed progressive increases in body weight and waist circumference. However, the US-CaCO_3_:Ce group exhibited a significant reduction in the growth rates of both body weight and waist circumference compared to the control group, starting in the second week. By the fourth week, the abdominal fat weight of the US-CaCO_3_:Ce group was only 71.04% of that of the control group (Fig. 6(c)). In obesity caused by excessive fat intake, adipocytes are ~ 10 times larger than the original size [43]. Further, we observed that compared to the control group, adipocytes in the fat section for the group subjected to US-CaCO_3_:Ce treatment had a smaller cell diameter (Figure S5). These results confirm that the combination of CaCO_3_:Ce with LIUS has a beneficial body-sculpting effect against localized obesity. Despite previous reports suggesting the potential cytotoxicity of certain rare-earth element nanoparticles (e.g., La_2_O_3_, Eu_2_O_3_, Dy_2_O_3_, and Yb_2_O_3_) [44], our findings indicate that the current strategy is safe. This conclusion is supported by the stable body temperature observed during treatment (Figure S6), normal biochemical and hematological profiles (Table S1), and the absence of pathological abnormalities in the histological sections of the heart, liver, spleen, lungs, and kidneys (Figure S7).

Sonoluminescent CaCO_3_:Ce microparticles were synthesized and simultaneously doped with Ce^3+^ and Ce^4+^ using an environmentally friendly method. The developed CaCO_3_:Ce can be combined with LIUS to generate ROS, CO_2_, and localized Ca^2+^, thereby efficiently reducing local fat for body sculpting and body weight management. The properties and efficacy of the developed US-CaCO_3_:Ce sonodynamic treatment were confirmed by comparison with those of US alone and free CaCO_3_:Ce while maintaining high biocompatibility and causing no systemic toxicity.

The designed strategy is compatible with current ultrasound devices used in hospitals and in the future, may have broad applications as a promising treatment for body sculpting.

## Conclusion

The sonosensitizer, Ce-doped CaCO_3_, was successfully synthesized and combined with LIUS for body sculpting in this study. The results illustrate that CaCO_3_:Ce exhibits excellent biocompatibility and demonstrates the capability to generate sufficient ROS in adipocytes for lipolysis. The animal experiments confirm that injecting the developed CaCO_3_:Ce under US irradiation in SD rats can significantly reduce the growth rate of body weight and the waistline as well as the accumulation of adipose tissue, which is indicated by the weight of subcutaneous fat. Biochemical and hematological tests and histological sections confirmed the safety of this approach.

In summary, the combination of Ce-doped CaCO_3_ and LIUS effectively inhibited adipogenesis and reduced fat tissue without inducing burning or charring of the skin and muscle tissue, thereby establishing that it is a safe and effective treatment for body sculpting.

## Materials and methods

### Cerium-doped calcium carbonate preparation

CaCO_3_:Ce was synthesized using a simple method at room temperature without the addition of organic solvents. In brief, 1.18 g of calcium nitrate (C2786, Sigma, USA) and 0.217 g cerium nitrate (011329, Alfa Aesar, UK) was dissolved in deionized water (50 mL). Then, 50 mL of 0.1 M sodium carbonate was added gently into the prepared calcium nitrate/cerium nitrate solution using a peristaltic pump at 5.0 rpm and stirred with a magnetic stirrer at 300 rpm at room temperature for 3 h. Then, the solution was centrifuged at 1300 rpm for 20 min (5500, Kubota, Japan). The precipitate was washed with ddH_2_O three times, and dried overnight in a freeze dryer (FDU-1100, EYELA, Japan) to obtain CaCO_3_:Ce. The synthesized particles were stored in a desiccator until subsequent use.

### Crystal structure identification

The crystal structure of CaCO_3_:Ce was identified using an XRD (MiniFlex II, Rigaku, Japan) with Cu Kα-II radiation at 30 kV and 15 mA, employing a scan rate of 4°/min within the range of 20 ~ 60°. The samples were sieved through a 230 mesh and pressed onto a sample holder with an area of 2 × 2 cm.

### Surface morphology and chemical composition analysis

The morphology and grain size of the synthesized particles were examined using SEM (TM-1000, Hitachi, Japan). The samples were mounted on the aluminum SEM sample stage and coated with a platinum film by sputtering using a physical vapor deposition method. The chemical compositions of the prepared samples were analyzed using EDS (JSM-5600, JEOL, Japan). The sample preparation process was similar as described above but coated with pyrolytic carbon instead of a platinum film. The accelerated X-ray beam energy was 20 kV.

### Electronic diffraction pattern examination

The morphology and electronic diffraction patterns of the developed particles were observed and analyzed using TEM (Tecnai G2 F20, FEI, USA). The CaCO_3_:Ce particles (5 mg) were dispersed in deionized water (1 mL) and homogenized using ultrasonic vibration for 15 min. Further, 20 µL of the homogenized particles were dropped onto a carbon-coated copper mesh and dried at room temperature in a desiccator. The accelerating voltage was 200 kV.

### Electronic states of the elements present on CaCO_3_

XPS was conducted using a spectrometer (Theta Probe, Thermo Scientific, USA) with mono-chromated Al *Kα* X-rays (*hv* = 1486.6 eV) as the excitation source. A standard lens mode and a spot size of 400 μm were used. The analyzer mode was CAE with a pass energy of 200.0 eV; the energy step size was 1 eV. The results were fitted using XPSPEAK software (version 4.1). The backgrounds were subtracted using the Shirley model. All binding energies were calibrated using the C (1s) carbon peak (284.5 eV).

### Analysis of particle size distribution

The particle-size distribution of CaCO_3_:Ce was analyzed using a zeta potential analyzer (Zetasizer Nano ZS, Malvern, UK). The sample was first suspended in deionized water and homogenized using ultrasonic vibration for 30 min. The homogenized suspension was transferred into a folded capillary zeta cell (DTS1070, Malvern, UK) and measured using dynamic light scattering (DLS).

### Evaluation of cell viability by WST-1 assay

Cell viability was assessed in L-929 cells (RM60091, Bioresource Collection and Research Center, Taiwan) using a WST-1 assay, following the ISO 10993-5 guideline. L-929 cells were cultured in α-MEM (11900-024, Gibco, USA) supplemented with 10% fetal bovine serum (FBS, A31606-02, Hyclone, USA) and 1% of antibiotic-antimycotic (Anti-anti, 15240-062, Gibco, USA) to constitute the complete medium. The extracted medium was used as an extraction vehicle for preparing the extracted sample solution. Each material (0.2 g of CaCO_3_:Ce, aluminum oxide (11028, Sigma, USA), and polyurethane film containing 0.1% zinc diethyldithiocarbamate (ZDEC, RM-A, Hatano Research Institute, Food and Drug Safety Center, Japan)) were immersed individually in 1 mL of complete medium at 37 °C under 5% CO_2_ for 24 h.

The cells were seeded into a 96-well culture plate at a cell density of 1 × 10^4^ per well and cultured at 37 °C under 5% CO_2_ for 24 h. The extracted media was separately cultured with previously seeded cells, and those were named and abbreviated as CaCO_3_:Ce group (CaCO_3_:Ce), negative control (N-control), and positive control (P-control), respectively. L-929 cells cultured in the complete medium were used as the control group, abbreviated as Control. After one day of incubation, the medium was removed and added to 100 µL of the complete medium containing 10% WST-1 reagent (11644807001, Roche, USA), which reacted at 37 °C under 5% CO_2_ for 30 min in a dark environment. The culture plate was mounted on a spectrophotometer (VersaMax™, Molecular Devices, Canada) and the absorbance at 450 nm was recorded to evaluate cell viability [25].

### Culture and differentiation method of 3T3-L1 cell

3T3-L1 pre-adipocytes cell line (60159, Bioresource Collection and Research Center, Taiwan) was seeded to a 12-well culture plate with a cell density of 1 × 10^4^ per well and cultured at 37 °C under 5% CO_2_ in Dulbecco modified Eagle medium (DMEM, high glucose, 12800-017, Gibco, USA) supplemented with 10% calf bovine serum (16170-078, Gibco, USA) and 1% 100X Anti-anti. After reaching cell confluence, the cells were further cultured for two more days to inhibit contact with the 3T3-L1 cells. The 3T3-L1 cells were cultured in an adipo-differentiated medium to convert cells into adipocytes, and the adipo-differentiated medium was DMEM supplemented with 10% FBS, 1% 100X anti-anti, 1mM dexamethasone (D4902, Sigma, USA), 0.2 M indomethacin (I7378, Sigma, USA), 0.1% insulin (I0516, Sigma, USA) and 0.25 M 3-isobutyl-1-methylxanthine (IBMX, I5879, Sigma, USA). The adipocytes were cultured in an adipocyte maintenance medium (DMEM supplemented with 10% FBS and 1% 100X Anti-anti), and the medium was refreshed every 3 days and the oil droplets were observed using a fluorescence microscope (TS-100, Nikon, Japan) stained with Nile red (N1142, Invitrogen, USA) [26].

### ROS generation

ROS generation in adipocytes induced by the synthesized CaCO_3_:Ce and exposed to LIUS was measured using CM-H_2_DCFDA (C6827, Invitrogen, USA) staining. 3T3-L1 cells were seeded into 96-well culture plates at a density of 1 × 10^4^ cells per well and differentiated into adipocytes. 100 µL of 0.5 mg/mL CaCO_3_:Ce in the culture medium was added into each well and cultured for 4 h. Then, it was exposed to LIUS from the bottom of the culture plate in degassed water using an US transducer with a diameter of 2.0 cm. The distance between the US transducer and the bottom of the cell culture plate was 5 mm. Ultrasound irradiation was performed using a function generator (33521 A, Agilent, USA) at a resonant frequency of 1.0 MHz and a duty cycle of 50%. Power amplification was used to generate a square wave with a negative pressure of 0.33 MPa and an intensity of 1.8 W/cm^2^ for 90 s [27]. After 1 h of incubation, the medium was changed to a 25µM CM-H_2_DCFDA solution and reacted at room temperature for 45 min. Fluorescence was excited at a wavelength of 493 nm, and the intensity of the emitted light was measured using a multi-label plate reader (EnSpire, PerkinElmer, USA) at a wavelength of 523 nm, which represents the ROS concentration.

The experiment was divided into four groups and abbreviated as follows: the cells were cultured in medium: (1) without CaCO_3_:Ce addition and no US applied (control); (2) application of LIUS without CaCO_3_:Ce addition (US); (3) with CaCO_3_:Ce addition but no exposure to LIUS (CaCO_3_:Ce); and (4) with CaCO_3_:Ce addition and exposure to LIUS (US-CaCO_3_:Ce).

### In vitro screening of adipocyte treated with synthesized CaCO_3_:Ce and LIUS by WST-1 assay and live/dead staining

The cell viability and cytotoxicity of adipocytes treated with synthesized CaCO_3_:Ce and exposed to LIUS were evaluated using the WST-1 assay and live/dead staining, respectively. The experiments were used as the first screening in vitro to determine body sculpting in vivo once adipose tissue was treated with the developed particles, followed by LIUS irradiation.

3T3-L1 cells were seeded in 12-well culture plates at a density of 6 × 10^4^ cells per well and differentiated into adipocytes. 0.5 mg/mL CaCO_3_:Ce was added to each well, cultured for 4 h, and exposed to LIUS. The cells were cultured for 1 h in an incubator. The medium was removed, and then, 900 µL of the culture medium and 100 µL WST-1 reagent was added, which was reacted at 37 °C under 5% CO_2_ for 1 h in the dark. The culture plate was mounted on a multi-label plate reader (EnSpire, PerkinElmer, USA), and the absorbance at 450 nm was recorded to evaluate cell viability.

In live/dead staining, the staining solution was prepared by mixing 3.3 µL of calcein AM (Ex/Em: 494/517 nm, C1430, Invitrogen, USA) and 1 µL of propidium iodide (PI, Ex/Em: 536/617 nm, P1304MP, Invitrogen, USA) reagents in 1 ml of phosphate buffered saline (PBS, pH 7.4). Adipocytes were treated with CaCO_3_:Ce and LIUS. After further culturing for 1 h, the medium was removed and 400 L of staining solution was added and allowed to react for 15 min at room temperature in the dark. The culture plate was mounted on a fluorescence microscope (TS100, Nikon, Japan) to label the living and dead cells with calcein AM (green) and propidium iodide (red), respectively, under proper excitation light.

### Animal experiments and surgical procedure

Ten-week-old male SD rats (body weight = 325 g) were used in this study. The rats were purchased from BioLASCO, Taiwan, and delivered to the Laboratory Animal Center, National Health Research Institutes, Taiwan, 7 days before the experiment began to accommodate them to the environment. One cage for each rat was placed in the experimental period with a controlled temperature and humidity of 22 °C and 55%, respectively, by toggling the light on and off every 12 h. The study protocol was approved by the Institutional Animal Care and Use Committee of the National Health Research Institute (NHRI-IACUC-108012).

A total of 2.5 mg of CaCO_3_:Ce was mixed with 1 mL of normal saline. Further, 100 µL of the mixture was injected into the fat tissue of the abdominal area of the SD rats once a week for four weeks. LIUS was applied to the area where CaCO_3_:Ce was injected and treated consecutively for 3 days every week for 4 weeks, for 90 s each time. The LIUS was generated by a function generator at a resonant frequency of 1.0 MHz, duty cycle of 50%, square wave with a negative pressure of 0.33 MPa, and intensity of 1.8 W/cm^2^.

The study was divided into four groups, with si × SD rats per group (*n* = 6). The groups were defined and abbreviated as follows: (1) rats without any treatment were categorized as the control group (Control); (2) rats that received LIUS without CaCO_3_:Ce injection on the abdominal fat tissue 3 days every week (US); (3) rats injected with CaCO_3_:Ce once a week without LIUS treatment (CaCO_3_:Ce); and (4) rats injected with CaCO_3_:Ce once a week and received US treatment consecutively for 3 days every week was the major experimental group (US-CaCO_3_:Ce).

The body weight, body temperature, weight, and waistline of the experimental rats were measured and recorded weekly. At the end of the experiment, the rats were sacrificed, and the blood was collected directly from the heart. Serum analysis was performed using a serology analyzer (DRI/CHEN NX-500 I, Fuji, Japan), and blood samples were analyzed using a hematology analyzer (BC-5000 VET, Mindray, China). Two analyses were performed to evaluate the safety of the newly developed lipolysis method in experimental animals. The results are summarized in the Supplementary Data. Finally, subcutaneous fat and organs were harvested for further analysis.

### Statistical analysis

All experiments were conducted at least in triplicate, and data are presented as mean ± standard deviation (SD). For in vitro experiments, statistical significance among multiple groups was assessed using one-way analysis of variance (ANOVA). For animal experiments, due to the small sample size (*n*<30), we applied the nonparametric Kruskal–Wallis test, as it does not assume normality and is more appropriate for small group comparisons. Statistical analyses were performed using GraphPad Prism version 8.4., and results were considered significant when the *p*-value < 0.05.

## Electronic supplementary material

Below is the link to the electronic supplementary material.

Supplementary Material 1

## Data Availability

No datasets were generated or analysed during the current study.
