# Supplementary material for: Cerium-doped calcium carbonate microparticles combined with low-intensity ultrasound for efficient sonodynamic therapy in body sculpting
Source: J Biol Eng. 2025 Apr 28;19:39. doi: 10.1186/s13036-025-00505-z (PMC12036128; doi:10.1186/s13036-025-00505-z)
Supplement: Supplementary file 1 — Supplementary Material 1 [file 13036_2025_505_MOESM1_ESM.pdf]

Supplementary material

Cerium-Doped Calcium Carbonate Microparticles Combined with Low-Intensity Ultrasound for Efficient Sonodynamic Therapy in Body Sculpting

Jhih-Ni Lin[1,2], Chih-Ying Chi[3], Yu-Ying Lin[2,4], Che-Yung Kuan[1,2], Chia-Tien Chang[2,4], Li-Ze Lin[5], I-Hsuan Yang[2,*], Feng-Huei Lin[1,2,*]

[1] Institute of Biomedical Engineering, College of Medicine and College of Engineering, National Taiwan University, Taipei, 106319, Taiwan

[2] Institute of Biomedical Engineering and Nanomedicine, National Health Research Institutes, Miaoli County, 350401, Taiwan

[3] Cardiovascular and Mitochondrial Related Disease Research Center, Hualien Tzu Chi Hospital, Buddhist Tzu Chi Medical Foundation, Hualien 970473, Taiwan

[4] Ph.D. Program in Tissue Engineering and Regenerative Medicine, National Chung Hsing University, Taichung, 402202, Taiwan

[5] Department of Materials Science and Engineering, National United University, Miaoli County, 360301, Taiwan

* These authors contributed equally to this work.

Corresponding Author:

Feng-Huei Lin:

Phone: +886-2-27320443
Fax: +886-2-23940049
E-mail: double@ntu.edu.tw

I-Hsuan Yang:

Phone: +886-37-206166 ext:37126,
Fax: +886-37-586440
E-mail: tony910028@gmail.com

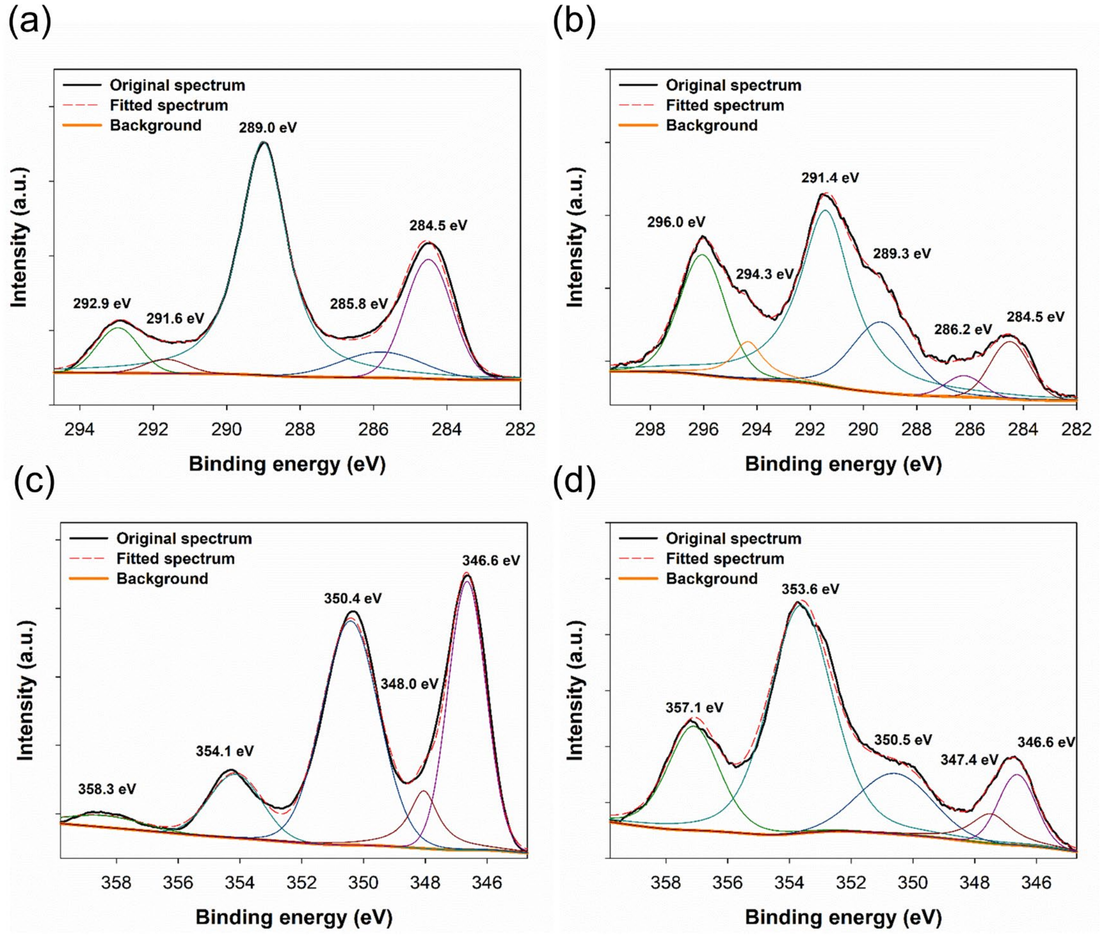

Figure S1. High-resolution XPS spectra of (A) C 1s for $CaCO_3$ (B) C 1s for $CaCO_3$:Ce, (C) Ca 2p for $CaCO_3$, and (D) Ca 2p for $CaCO_3$:Ce.

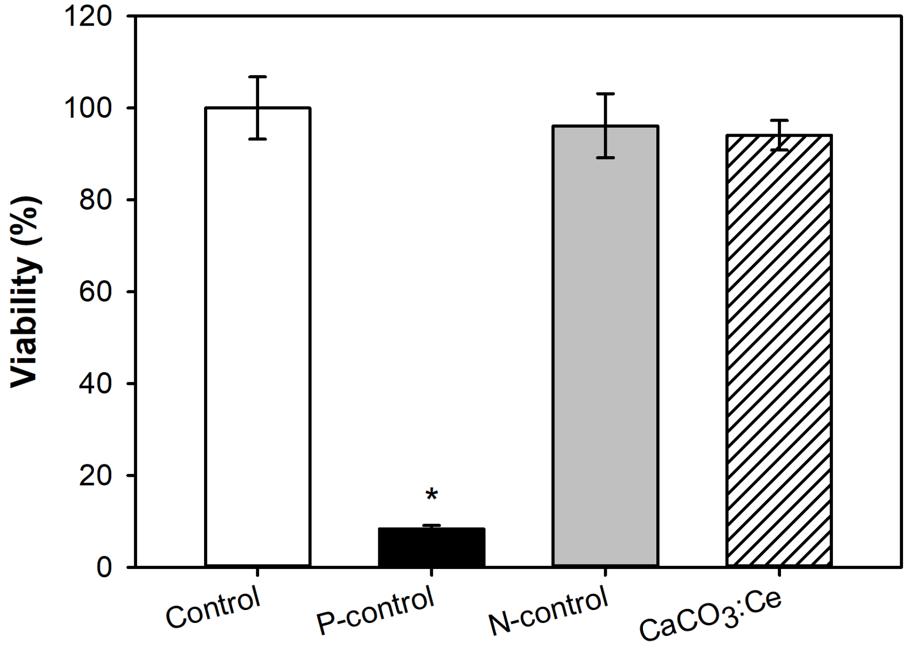

Figure S2. The evaluation of cell viability of synthesized $CaCO_3$:Ce by WST-1 assay. *$p < 0.05$.

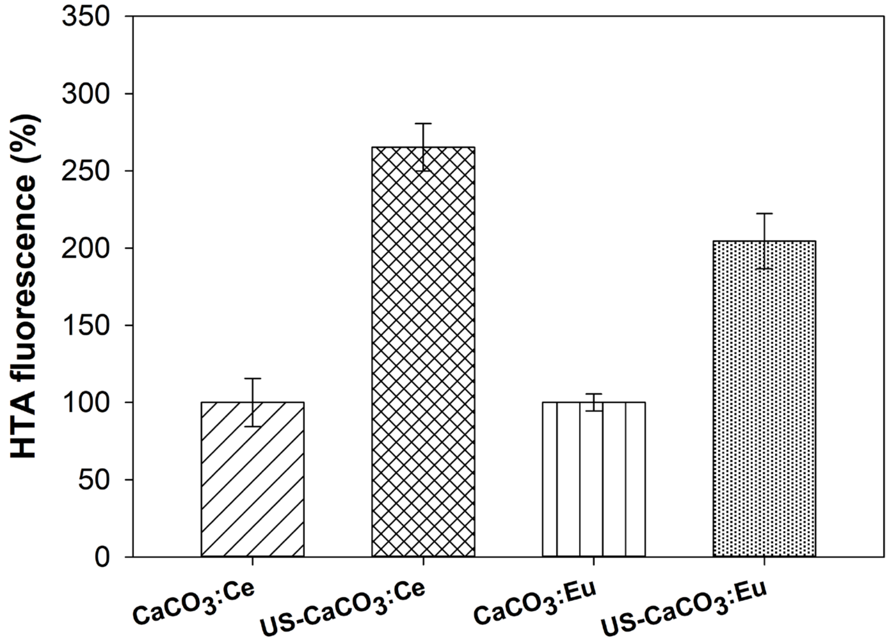

Figure S3. The HTA production of synthesized particles with LIUS irradiation.

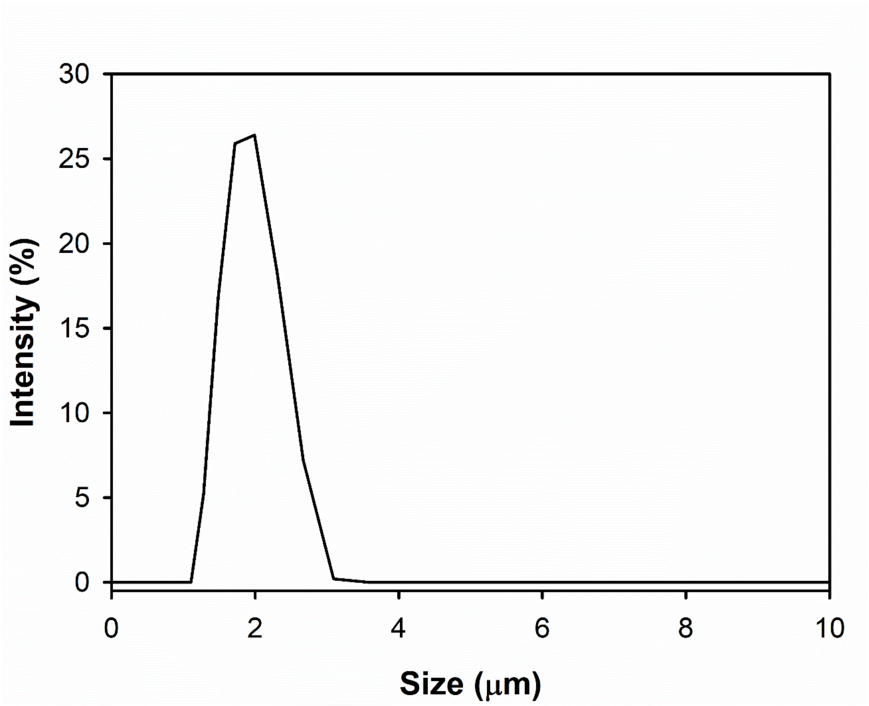

Figure S4. Size distribution profile of $CaCO_3$:Ce. The measured mean particle size was 1.77 μm, and the polydispersity index (PDI) was 0.22, indicating a relatively uniform size distribution.

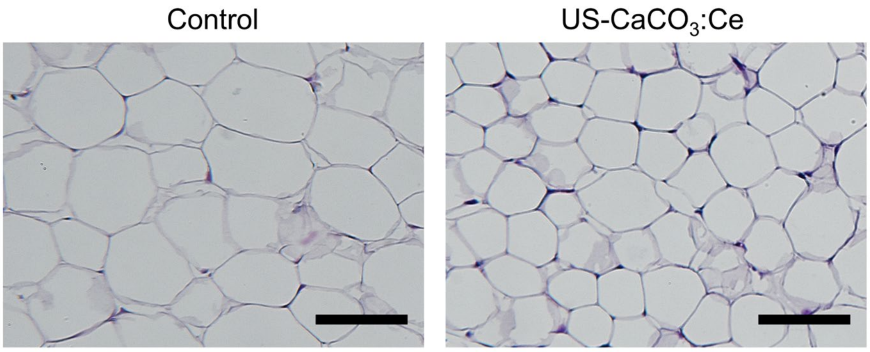

Figure S5. Histological sections with H&E stain on subcutaneous fat tissue (scale bar: 50 μm).

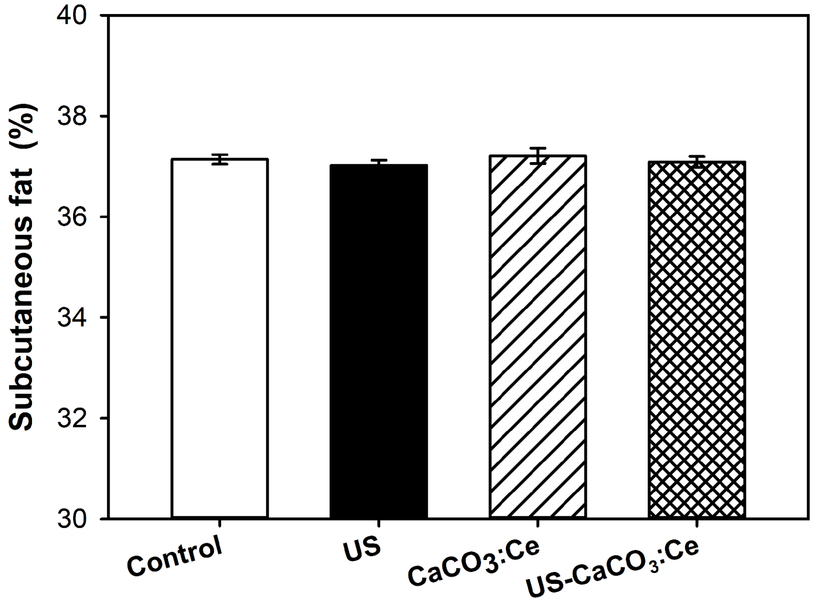

Figure S6. Measurement of body temperature on SD rats.

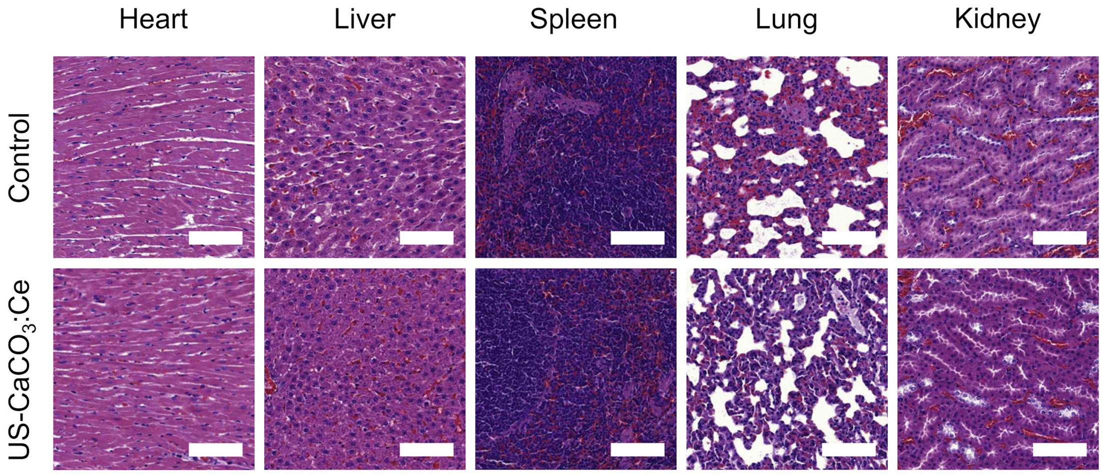

Figure S7. Histological sectioning with Hematoxylin and eosin (H&E) stain (scale bar: 100 μm).

Table S1. Biochemical and hematological tests.

Variables	Control (n=5) *	US-$CaCO_3$:Ce (n=6)
WBC (K/μL)	7.42 ± 4.6	7.37 ± 5.76
NE (%)	19.83 ± 3.41	21.34 ± 2.66
LY (%)	73.02 ± 1.66	73.14 ± 2.03
MO (%)	4.22 ± 0.93	4.46 ± 2.03
EO (%)	0.89 ± 0.77	0.74 ± 0.83
BA (%)	0.39 ± 0.43	0.32 ± 0.39
RBC (M/μL)	6.96 ± 1.13	7.29 ± 0.81
HGB (g/dL)	13.53 ± 0.67	14.1 ± 0.82
HCT (%)	42.58 ± 3.24	43.8 ± 2.98
PLT (K/μL)	794.73 ± 279.49	763.67 ± 385.22
AST (U/L)	187 ± 58.21	164.67 ± 68.68
ALT (U/L)	48.04 ± 8.79	49.67 ± 6.43
BUN (mg/dL)	21.5 ± 3.06	22.23 ± 1.66
CRE (mg/dL)	0.35 ± 0.08	0.25 ± 0.06
UA (mg/dL)	1.41 ± 1.10	1.23 ± 0.85
TG (mg/dL)	149.97 ± 34.24	152.33 ± 33.32
TCHO (mg/dL)	76.25 ± 9.13	60.33 ± 8.08
Ca (mg/dL)	9.45 ± 0.98	8.97 ± 0.64

WBC- White blood cell; NE- Neutrophil; LY- Lymphocyte; MO- Monocyte; EO- Eosinophil; BA- Basophil; RBC- Red blood cell; HGB- Hemoglobin; HCT- Hematocrit; PLT- Platelet; AST - Aspartate aminotransferase; ALT - Alanine aminotransferase; BUN - Blood urea nitrogen; CRE- Creatinine; UA- Uric acid; TG- Triglycerides; TC- Total cholesterol; Ca- Calcium.

(n=5) *: One data point in the control group was likely an artifact caused by partial blood coagulation during sample collection. To ensure data accuracy, we removed this compromised data point and analyzed the results.
